# Performance evaluation of laboratory professionals on malaria microscopy in Hawassa Town, Southern Ethiopia

**DOI:** 10.1186/1756-0500-7-839

**Published:** 2014-11-25

**Authors:** Freshwork Ayalew, Birkneh Tilahun, Bineyam Taye

**Affiliations:** College of Medicine and Health Sciences, Hawassa University, Hawassa, Ethiopia; School of Laboratory Medical Sciences, College of Health Sciences, Addis Ababa University, Addis Ababa, Ethiopia; School of Public Health, College of Health Sciences, Addis Ababa University, Addis Ababa, Ethiopia

**Keywords:** Malaria microscopy, Performance, Laboratory professionals, Ethiopia

## Abstract

**Background:**

Microscopic diagnosis of Giemsa stained thick and thin blood films by skilled microscopists has remained the standard laboratory method for the diagnosis of malaria. However, detection and identification of malaria parasites require well trained laboratory personnel.

The objective of the study was to evaluate the performance of laboratory technologists and technicians in detecting and identifying malaria parasites in Hawassa town, Southern Ethiopia.

**Methods:**

A cross-sectional study design was employed among a total of 80 laboratory professionals working in public and private health facilities. A standardized pre-validated slide panel and questionnaires were distributed to laboratory professionals working at eleven health facilities in Hawassa town, Southern Ethiopia. The panels included ten slides for diagnosis, [slide1:*P.falciparum,* 104/μl; slide 2:*P.falciparum,* 53404/μl; slide 3 and 4: mixed infection (both *P. falciparum* and *P. vivax*); slide 5:*P.vivax,* 23503/μl; slide 6:*P.vivax*, 400/μl; and slides 7, 8, 9 and 10: negative slides]. Participants were asked to return the responses which were compared with expert microscopist. Agreement in detecting and identifying malaria parasites between participants and expert microscopists was estimated using the Kappa score.

**Results:**

The mean age of the participants was 27 (SD = 4.1) years. More than half of the participants (56.9%) were female. Fourteen (19.4%) of the participants correctly reported all the ten distributed slides, whereas 58(80.6%) missed at least one slide. Overall, the sensitivity and specificity of participants in detection of malaria parasites were 82% and 96.5% respectively. The overall agreement between participants and reference readers on detection of malaria parasite was 88% (Kappa = 0.76) while on identification of malaria species was 74.3% (kappa = 0.63). Lower agreement on detection and identification of slides with low parasitic density and mixed infection were observed. Agreement was relatively lower for government health centers (69%; kappa = 0.56). None of the participants reported parasitic load per micro liter method.

**Conclusion:**

Agreement of the participants with expert microscopist in the detection of malaria parasites was better than agreement in the identification of different species of malaria. Poor agreement was reported in detection of parasites at a low density and mixed infections.

## Background

Malaria is the leading public health concern in the Southern Nation Nationalities and People Region (SNNPR) among the regional states of Ethiopia. Epidemiological and ecological data of the region indicate that more than 75% of the area of this region is malarious [1, 2].

Early diagnosis of malaria is a basis for the management of malaria, and key to reducing malaria related mortality and morbidity. Demonstration of the presence of malaria parasites under microscopy prior to treatment with anti-malarial drugs is fundamental to this goal since clinical diagnosis has a poor accuracy and leads to over-diagnosis and increases the risk of anti-malarial drug resistance [2, 3].

Parasites of the Plasmodium species can be detected in stained blood smear by light microscopy. Microscopy is a simple and cost effective for detecting and identifying different stages of Plasmodium species in peripheral blood smear. However, this method is time-consuming and requires expertise especially in patients with low level parasitemia [4]. In many developing countries, microscopists are insufficiently trained, poorly supervised and burdened with a high work load [5].

In Ethiopia, laboratory diagnosis of malaria used to be performed by malaria microscopists working in malaria control offices. However, after decentralization and integration of independent programs, general laboratory technicians are in charge of malaria diagnosis services at health care facilities [6, 7]. However, data are lacking on performance of laboratory professionals’ in detection and identification of malaria parasites in the study area.

The current study aimed to evaluate the performance of laboratory professionals in detecting and identifying different stages of Plasmodium species using light microscopy.

## Methods

### Study setting and population

A cross sectional study design was conducted from November 2013 to January 2014 in Hawassa town. Hawassa is the capital of the Southern Nations Nationalities and Peoples Region (SNNPR), located at 275 km from Addis Ababa, the capital of Ethiopia. The altitude is 1697 m above sea level, the mean annual temperature is 20.9 Celsius, and the annual rainfall is 997.6 mm. According to 2007 census conducted by the Central Statistical Agency of Ethiopia, the total population of Hawassa Town was 258,808 with almost 1:1 male to female ratio. Although malaria occurs throughout the year, the peak malaria transmission occurs during the months of August to December [8]. There are two government and three private hospitals and six government health centers. The laboratory in these facilities provided malaria microscopy in daily basis.

A total of 80 laboratory professionals working in 11 health facilities at Hawassa Town were involved in the current study.

### Panel slide preparation and distribution

Four milliliters of whole blood were collected from acutely febrile patients who attended the Adama malaria control laboratory after obtaining informed consent. The blood samples were transferred into separate EDTA containing glass tubes. They were then processed within one hour of collection to preserve leukocyte and parasite morphology. Blood samples were also collected from malaria negative persons. The controls were healthy individuals who consented to give sample. The negative blood samples were also transferred to an EDTA containing tube for further use of negative slide set preparation. All cases positive for *P. falciparum* were treated with artemether-lumefantrine, whereas cases with *P. vivax* were treated with chloroquine [9].

Two expert microscopists who have been pre-qualified and certified by WHO as Level I Expert Microscopists prepared and validated the slide panels at Adama Malaria Control Laboratory.

In an attempt to standardize the preparation of thick blood films, we took 6 μl of blood with an automatic pipette, and evenly spread the blood on a microscopic slide over an area of 11 × 12 mm. This was done by placing the slide on a template marking the exact area. In addition, we also took 2 μl of blood for the preparation of a thin blood film in each slide. Each slide with thick and thin film was dried overnight, and the thin film was fixed by dipping in absolute methanol. Then, they were stained with 3% Giemsa for 30–45 minutes [10]. The blood films were mounted (by polymount) and cover slipped to increase their shelf life [11]. Once the Giemsa-stained slides became dry, they were stored in a clean slide box. After preparation of the panels was completed, the two expert microscopists arranged the slides in ten sets. The quality of the slides was checked before packing the sets, and the panels were validated before dispatching.

### Slide panel characteristics

Slides were prepared based on the species present in the Hawassa Region: *P. vivax*; *P. falciparum*; and mixed slides (*P. falciparum and P. vivax*). Each panel of slides included six slides with different parasite densities (low and high density) and four negative slides. The total number of slides per panel was ten. Groups of uniform panels with respect to the characteristics of the positive (species and level of parasitemia) and negative slides were used so that the results of the evaluation by different laboratories could be compared.

The two malaria microscopy experts interpreted the blood smears using three diagnostic criteria: 1) The presence or absence of malaria parasites; 2) Identification of the species of parasites; 3) Quantification of parasitic load for each species. “Load” was defined as number of parasites per 200 white blood cells in high power thick fields and multiplied by a standard multiplier of 8,000 WBC/μl of blood [12].

Slides were considered negative if no malaria parasites were seen in 100 × magnification oil immersion fields. After validation of the slides was completed, the slides were arranged in ten sets and then packed for distribution to the participant laboratories. The reporting formats and instruction letters were packed separately [13].

### Administration of the slide panel test

After validation, ten Giemsa stained thin and thick blood films with questionnaires were delivered to laboratory professionals working at eleven health facilities in Hawassa town. The panels included ten slides for diagnosis, [slide1:*P. falciparum,* 104/μl; slide 2:*P.falciparum,* 53404/μl; slide 3 and 4: mixed infection (both *P. falciparum* and *P. vivax*); slide 5:*P.vivax,* 23503/μl; slide 6:*P.vivax*, 400/μl; and slides 7, 8, 9 and 10: negative slides].

For slide examination by professionals, 10 minutes per slide was allocated [13]. It was performed individually and in two sessions. Randomly, five slides of a panel of 10 slides were examined on the first day and the remaining five on the next day. Each of the participants spent 50 minutes per session on the day of their convenience. The data collector retrieved the slides after each participant completed the tests.

### Questionnaire

A structured questionnaire including information on the participating facilities and professionals was distributed. The questionnaire had sub-components like: the socio-demographic characteristics, educational background, service, in service training and routine practice of the professionals, and type and quality of microscope. The questionnaire was first pre-tested on 5% of the participating professionals and was improved as needed before the beginning of the study.

### Data management and quality assurance

Data quality was assured through use of standardized data collection materials, pretesting of the questionnaires, and intensive supervision during data collection by the principal investigator.

### Statistical analysis

Data were entered, cleaned and analyzed using SPSS for windows version 16. Level of performance in detection of malaria parasite was compared with independent variables. Association was taken as significant at P < 0.05. Mean, standard deviation, chi-square (for categorical data), sensitivity, specificity, proportion of errors, percent agreement, and kappa score were calculated to assess laboratory professionals’ performance in detecting and identifying different stages of Plasmodium species using light microscopy.

The result of the microscopic diagnosis of malaria reported by the participants was evaluated using various parameters. Sensitivity was determined as the ability of participants to diagnose positive blood films; whereas, specificity was calculated for their ability to diagnose negative blood films.

“Major error” was defined as incorrect diagnosis of malaria, i.e. reporting “negative” in the case of a Plasmodium-positive sample and reporting “positive” in Plasmodium negative samples; and falsely reporting non-falciparum species in the case of *P. falciparum parasitemia.* [14]. “Minor error” was defined as identification error of *P.vivax* and of a mixed infection (reporting single infection in case of mixed parasites) [15]. The distinction between a minor and a major error was based on the effect the error could potentially have for the patient's diagnosis and clinical management [16].

Based on WHO recommendations, participants were classified as: “In training”- when the agreement with the reference reader in detection of malaria parasite was less than 70%; “Competent”- when the agreement was greater than or equal to 70% but less than 80; “Reference”- when the agreement was greater than or equal to 80% but less 90%; and “Expert” -when the agreement was greater than or equal to 90% [17].

Inter-rater agreement is the degree of agreement between two reference readers. It is calculated by computing the sum of true positives and true negatives and then divided by the total.

### Ethical consideration

The study was approved by departmental research and ethics review committee (DRERC) of the Addis Ababa University, Department of Medical Laboratory Science. Official letter was written to the participating facilities. Consent forms were prepared in English to be read and signed (if agreed) by the participating health professionals and for blood donors. Interpreters were assigned for non-English speakers among donors. Information obtained about the laboratory professionals from the questionnaires, data capturing formats and the slides were kept totally anonymous. Participants had the right not to participate or to withdraw from the study at any time.

## Result

A total of 72 out of 80 laboratory professionals responded to the questionnaires with a response rate of 90%. Thirty-two (44.4%) of the participants were from two government hospitals, 9(12.5%) were from three private hospitals and 31(43.1%) were from six government health centers. The mean age of the participants was 27 (SD = 4.1) years and 41 (56.9%) were female (Table 1).

**Table 1:** **Demographic characteristics of laboratory professionals, Hawassa Town, Southern Ethiopia (N = 72)**

Variables	Number	Percent (%)
**Age in year**		
20-30	59	81.9%
31-40	12	16.7
>41	1	1.4
**Sex**		
Male	31	43.1
Female	41	56.9
**Place of work**		
- Government hospitals	32	44.4
- Private hospitals	9	12.5
- Government health centers	31	43.1
**Educational status**		
Diploma	44	61.1
Degree	19	26.4
Advance standing	9	12.5
**Experience in routine malaria diagnosis**		
<2 years	16	22.2
≥2 years	56	77.8
**Participation in in-service training about malaria microscopy**		
Yes	23	31.9
No	49	68.1
**Frequency of participation in malaria microscopy training**		
Once	18	78.3
Twice	3	13
3 times	2	8.7
**Have you ever been supervised by regional or national laboratories**		
Yes	30	41.7
No	42	58.3
**Do you use RDT for malaria diagnosis**		
Yes	8	11.1
No	64	88.9

Half of the participants examined more than ten blood film slides per day and 51(70.8%) of the participants used both thick and thin blood films for detection and identification of malaria parasites. On the other hand, a considerable number of participants (23.6%) examined thick films only. More than half (56.9%) of the participants had experience in reporting parasite count using a grading system. There were no problems with the functionality of microscopes and accessibility of reagents in any of the laboratories.

**Figure 1:**
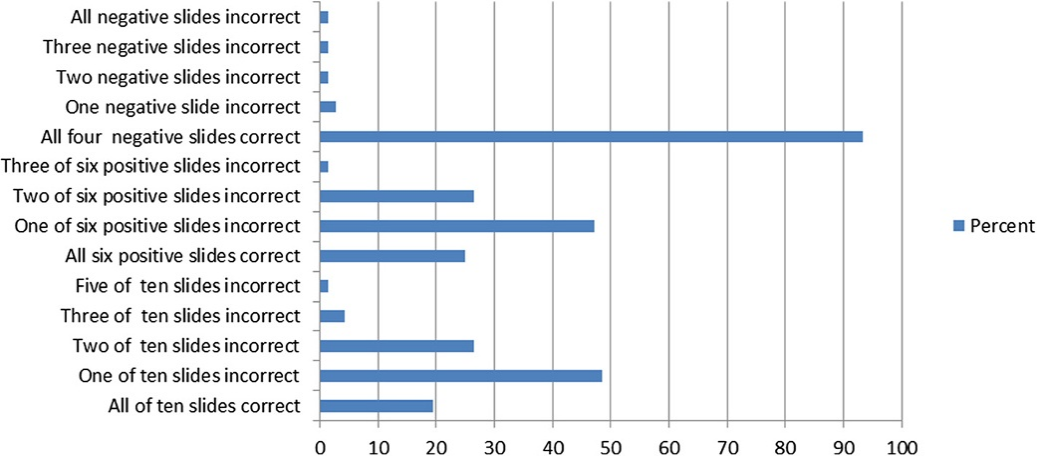
**Distribution of error in detection of malaria parasites among participants, Hawassa Town, Southern Ethiopia (n = 72), 2014.**

Of 72 participants, 14(19.4%) correctly interpreted all ten distributed slides, and 58(80.6%) missed at least one slide. Eighteen (25%) of the participants reported correct results of all positive samples and 67(93.3%) of participants correctly reported all four negative slides (Figure 1).

There was no statistically significant association between the proportion of errors made by the participants in the detection of malaria parasites and their sex, experience, number of slides examined per day, in-service training, and supervision (Table 2).

**Table 2:** **Relationship between reporting errors in detection with selected demographic characteristics of participants, Hawassa Town, Southern Ethiopia, 2014**

Variable	All ten slides correct (%)	At least one error (%)	Chi squire	Degree of freedom	P- value
**Sex**					
Male	6(19.4)	25(80.6)	0.072	1	0.8
Female	9(22.0)	32(78.0)			
**Experience**					
< 2 year’s	3(18.7)	13(81.3)	0.205	2	0.9
≥2 years	11(19.6)	45(80.4)			
**Number of slides**					
**Examined per day**					
<5	3(25.0)	9(75.0)	0.752	2	0.7
5-10	6(25.0)	18(75.0)			
>10	6(16.7)	30(83.3)			
**In service training**					
Yes	3(13.0)	20(87.0)	1.243	1	0.3
No	12(24.5)	37(75.5)			
**Supervision**					
Yes	4(13.3)	26(86.7)	1.754	1	0.2
No	11(26.2)	31(73.8)			

Overall, the sensitivity and specificity of participants in detecting malaria parasites as compared to the two expert malaria microscopists were 82% and 96.5% respectively. Agreement with expert microscopists was 88% (Kappa = 0.76) on detection of malaria parasite. Participants also had 80.8% positive agreement for the six positive slides and 77.5% negative agreement for the four negative slides. Comparison across institutions showed lower sensitivity (81.0%) in those working in government health centers with an agreement of 87.0% (Kappa = 0.74) with reference readers (Table 3).

**Table 3:** **Overall sensitivity, specificity and agreement of participants in detecting malaria parasite by health institution based on total number of observations**

Health institution	Participant reader	Reference reader						
		Positive	Negative	Total	Sensitivity	Specificity	Agreement	Kappa
Government hospitals	Positive	160	2	162	83%	98%	89%	0.78
	Negative	32	126	156				
	Total	192	128	320				
Private hospitals	Positive	46	3	49	85%	92%	88%	0.76
	Negative	8	33	41				
	Total	54	36	90				
Government health centers	Positive	150	5	155	81%	96%	87%	0.74
	Negative	36	119	155				
	Total	186	124	310				

Overall agreement between participants and the two expert malaria microscopists on detection and identification was 74.3% (Kappa = 0.63). Agreement in detection (88%) was much higher than in identification (74.3%) of malaria species. Comparison across institutions showed that agreement in identification was higher in government hospitals (79%) (Kappa = 0.70). The lowest agreement on identification was found among participants working in government health centers with an agreement of 69% with reference readers (Kappa = 0.56) (Table 4). For both *P.vivax* and *P. falciparum,* worst agreement was found for slides with low parasite density (Table 5).

**Table 4:** **Agreement of participants in identification of malaria species by health facility (N = 72)**

Facility	Participant reader	Reference reader						
		Negative	*P. falciparum*	*P. vivax*	Mixed	Total	Agreement	Kappa
Gov’t^*a*^ hospitals	Negative	125	23	9	0	157	79%	0.70
	*P. falciparum*	1	35	1	25	62		
	*P. vivax*	1	4	54	1	60		
	Mixed	0	2	0	38	40		
	Total	127	64	64	64	319		
Private hospitals	Negative	33	6	2	0	41	74%	0.64
	*P. falciparum*	2	11	1	3	17		
	*P. vivax*	1	1	15	6	23		
	Mixed	0	2	0	9	11		
	Total	36	20	18	18	92		
Gov’t health centers	Negative	119	21	15	0	155	69%	0.56
	*P. falciparum*	4	35	7	28	74		
	*P. vivax*	1	6	40	14	61		
	Mixed	0	0	0	20	20		
	Total	124	62	62	62	310		

^a^Government.

**Table 5:** **Agreement of participants with reference readers for negative and positive slides based on experience**

Agreement of participants with reference readers		
Slide characteristics	Participants <2 years of experience	Participants ≥2 years of experience
Negative slides	87.5%	94.6%
*P.vivax* at low density	50%	64.3%
*P.falciparum* at low density	12.5%	23.2%
Mixed*	25%	37.5%

*Both *P.vivax and P.falciparum.*

None of the participants reported parasite density on each of malaria positive slides using parasite per microliter against white blood cells on thick blood film though it was one of the required parameters to be reported.

Major errors were reported by 79.2% and 12.5% of participants for slide 1 and 2 of *P. falciparum* positive slides, respectively. On the other hand, 55.6% and 51.4% of participants reported minor errors on slides 3 and 4 of mixed infections. On slides 5 and 6 (*P. vivax* positive), 1.4% and 34.3% reported major errors; while, 9.7% and 4.2% reported minor errors respectively. A small proportion (1.4%, 2.8%, 4.2% and 5.6%) of participants made major errors (reported false positive result) on the respective negative slides 7, 8, 9 and 10 (Table 6).

**Table 6:** **Proportion of major and minor errors made by participants in reporting each of the ten slides in the panel (n = 72)**

I Positive slides					
	Correct (%)	Major error (%)			Minor error (%)
Slide #		Detection error (%)	Identification error of *P. f* ^*#*^(%)	Total (%)	Identification error of (mixed and P. v*) (%)
1	15(20.8)	48(66.7)	9(12.5)	57(79.2)	-
2	63(87.5)	2(2.8)	7(9.7)	9(12.5)	-
3	32(44.4)	-	-	-	40(55.6)
4	35(48.6)	-	-	-	37(51.4)
5	64(88)	1(1.4)	-	1(1.4)	7(9.7)
6	44(61.1)	25(34.3)	-	25(34.3)	3(4.2)
**II Negative slides**					
**Slide #**	**Correct (%)**	**Detection error (%)**	**ID** ^**$**^ **error**	**Total (%)**	**Minor error**
7	71(98.6)	1(1.4)	-	1(1.4)	-
8	70(97.2)	2(2.8)	-	2(2.8)	-
9	69(95.8)	3(4.2)	-	3(4.2)	-
10	68(94.4)	4(5.6)	-	4(5.6)	-

^#^ P. falciparum *P. vivax ^*$*^Identification.

Among mixed infection slides, 44.4% and 48.6% of participants reported correct results on slide 3 and 4 respectively. However, 55.6% and 51.4% reported minor errors for the respective slides. Most cases of mixed infection were reported as *P. falciparum*: 26(81.3%) for slide 3 and 30(85.7%) for slide 4.

Based on the WHO grading system, 17(23.6%) of the participants were rated as ‘in training”, 23(31.9%) as “competent”, 14(19.4%) as “reference”, and 18(25%) as “expert” level. Among 56 participants who had two or more years of experience in microscopic diagnosis of malaria, 11(19.6%) were classified as ‘in training’, 18(32.1%) were competent and 13 (23.2%) were “reference” and 14(25%) reached agreement on an “expert” level. Among participants who had less than two years of experience, 6 (37.5%) were classified in the group of “in training”, 5(31.3%) were considered “competent”, 1(6.3%) achieved at “reference” and 4(25%) reached to “expert”. However, there was no statistically significant difference in level of agreement based on experience.

## Discussion

Microscopy of Giemsa stained thick and thin blood films is the standard for the diagnosis of malaria. However, interpretation of results requires professional expertise especially when the parasite density is low. In the current study, agreement between reference readers and participants in the detection of malaria parasites was 88% and in the identification of different species of malaria was 74.3%. Fourteen (19.4%) of participants correctly reported all the ten distributed slides, and 58(80.6%) missed at least one slide.

Overall, the sensitivity and specificity of laboratory professionals in detecting malaria parasites were 82% and 96.5% respectively. These findings of low sensitivity and relatively high specificity were in agreement with a study conducted in Zambia (88% and 97% respectively) [18]. Low sensitivity in detection of malaria parasites indicates that there were many false negative results, i.e. missed diagnoses of true infections. This can lead to delayed treatment, development of serious complications and death or exposure to unnecessary treatment with other (not anti—Malaria) drugs.

However, our findings of sensitivity and specificity were higher than in other studies conducted in Tanzania, Ethiopia and Haiti, where sensitivity and specificity of detection of malaria parasites by laboratory professionals were (74.5%, 59%) in Tanzania, (78.5%, 73.7%) in Ethiopia and (66.3%, 88.6%) in Haiti [19–21].

Our finding of an overall agreement on detection of malaria parasites with reference readers was 0.67 which is defined as ‘substantial’ based on the Kappa index interpretation by Landis and Koch [22]. The overall agreement in the current study was only slightly higher than in a study by Clendennen et al. (Kappa = 0.61) [23]. It was much better than reported in a study from four laboratories in Mpumalanga province, South Africa, which showed a worryingly high level of disagreement (kappa = 0.11) [24]. Overall, agreement in identification of different species of malaria in the current study was 0.63 which is higher than the finding reported in North Gondar (kappa = 0.41) [21].

The frequency of errors in the diagnosis of *P. falciparum* in the present study was higher than reported from the USA (3.4% error for species of *P. falciparum*), Hong Kong (5% failure rate in species diagnosis of *P. falciparum*), Canadia (22% errors in the species diagnosis of *P. falciparum*), UK (21% errors in identification of *P. falciparum*) and Democratic Republic of Congo (34.9% errors in identification of *P. falciparum*) [14, 15, 25–27].

Our findings show that false positive results were reported by 6.9% of participants. This finding is higher than the 2.0% false positive result reported in the Canadian study [26]. However, it is lower than 7.8% false positive result reported in USA [25] and 24.6% false positive result reported in Democratic Republic of Congo [14]. These false positive results suggest that participants often incorrectly report the presence of parasites; this could lead to unnecessary treatment or a delayed diagnosis of the true cause of illness and distract the clinician from considering other causes of fever and disease.

In the current study, minor errors made on each of two mixed slides were less frequent than reported in studies from the UK where the proportion of minor error was 71% [27]. However, they were more frequent than in studies reported from the Peruvian Amazon (25%) [28]. Our findings showed that most cases of mixed infections were reported as *P. falciparum* in contrast to the study conducted in the Peruvian Amazon where most cases of mixed infections were reported as negative or *P. vivax* [28]. This may be due to the fact that some participants did not spend sufficient time to examine the slides, or possibly due to lack of awareness of the possibility of the presence of more than one species in blood films.

Participants who had two or more years of experience had poorer agreement for slides with *P. falciparum* at a low level of parasitemia (23.2%). This finding is much lower than for slides of *P. falciparum* with low parasite density reported from the Peruvian Amazon [28]. This indicates the need of stringent refreshment training with special emphasis for detection at a low level of parasitemia. The challenge in detection and identification of malaria parasites was also reported in a study conducted in Cambodia, where there were problems even among expert level microscopists in the identification of malaria parasites on slides with low level parasitemia [11].

The limitation of this study is that we only used proficiency testing slides using unknown panels to evaluate the skill of laboratory professionals under optimal conditions, rather than routine or day to day performance in the diagnosis of malaria. Furthermore, we did not evaluate the performance of the laboratory personnel in regards to the preparation of the smears and the staining of blood films for malaria diagnosis.

## Conclusion

Participants had low sensitivity and relatively high specificity in the detection of malaria parasites. Agreement of the participants with expert microscopist in the detection of malaria parasites was better than agreement in the identification of different species of malaria. Poor agreement was reported in the detection of parasites at a low density level and mixed infections. Participants from government health centers were found to have lower performances in identification of malaria parasites.
